# Nitrate–Nitrite Interplay in the Nitrogen Biocycle

**DOI:** 10.3390/molecules30143023

**Published:** 2025-07-18

**Authors:** Biplab K. Maiti, Isabel Moura, José J. G. Moura

**Affiliations:** 1 Department of Chemistry, School of Sciences, Cluster University of Jammu, Jammu 180001, India; 2 LAQV, NOVA School of Sciences and Technology, Campus de Caparica, 2829-516 Caparica, Portugal; isabelmoura@fct.unl.pt

**Keywords:** nitrogen-biocycle, nitrate reductase, nitrite oxidoreductase, mo-dependent enzymes

## Abstract

The nitrogen cycle (N-cycle) is a cornerstone of global biogeochemistry, regulating nitrogen availability and affecting atmospheric chemistry, agricultural productivity, and ecological balance. Central to this cycle is the reversible interplay between nitrate (NO_3_^−^) and nitrite (NO_2_^−^), mediated by molybdenum-dependent enzymes—Nitrate reductases (NARs) and Nitrite oxidoreductases (NXRs). Despite catalyzing opposite reactions, these enzymes exhibit remarkable structural and mechanistic similarities. This review aims to elucidate the molecular underpinnings of nitrate reduction and nitrite oxidation by dissecting their enzymatic architectures, redox mechanisms, and evolutionary relationships. By focusing on recent structural, spectroscopic, and thermodynamic data, we explore how these two enzyme families represent “two sides of the same coin” in microbial nitrogen metabolism. Special emphasis is placed on the role of oxygen atom transfer (OAT) as a unifying mechanistic principle, the influence of environmental redox conditions, and the emerging evidence of bidirectional catalytic potential. Understanding this dynamic enzymatic interconversion provides insight into the flexibility and resilience of nitrogen-transforming pathways, with implications for environmental management, biotechnology, and synthetic biology.

## 1. Introduction

The nitrogen (N) cycle is a natural process by which nitrogen moves through the atmosphere, soil, water, plants, animals, and microbes [1,2,3,4,5]. The excessive use of fertilizers in agriculture, accumulating nitrate (NO_3_^−^) and releasing nitrous oxide (N_2_O), are inherently problematic issues [1,2,3,4,5,6,7,8,9,10]. The N-cycle includes several key stages: nitrogen fixation is the step where an atmospheric nitrogen molecule (N_2_) is converted into ammonia (NH_3_) [9] or related compounds by bacteria in the soil or through industrial processes and nitrification is the step where nitrite (NO_2_^−^) oxidation occurs (and NO_3_^−^ reduction), a key process in the N-cycle, which is essential for maintaining nitrogen balance in ecosystems, making nitrogen available to plants [1,2,3,4,5,6,7,8,9,10,11]. Nitrite-oxidizing bacteria catalyze the oxidation of NO_2_^−^ to NO_3_^−^ using molybdenum-containing nitrite oxidoreductase (NXR) [6]. Nitrite oxidation seems to be a principal source of nitrate under aerobic conditions [12]. Assimilation is the step that absorbs nitrates and ammonia to synthesize proteins and other organic molecules. Ammonification (decomposition) breaks down dead organisms and waste products, returning ammonia to the soil. Denitrification is a very specialized avenue, where bacteria complete the cycle, bringing nitrate back to nitrogen gas, and releasing it into the atmosphere [6,13,14,15,16,17].

In parallel to nitrification, two processes have been added to the complexity of the N-cycle: Anammox (Anaerobic Ammonium Oxidation), revealed as a key process in the nitrogen cycle where bacteria convert ammonium and nitrite directly into nitrogen gas, removing nitrogen from ecosystems, and Comammox (Complete Ammonia Oxidation), which refers to bacteria that can oxidize NH_4_^+^ to NO_3_^−^ in a single step, rather than in two separate steps by different organisms (as seen in traditional nitrification) [18].

**Figure 1:**
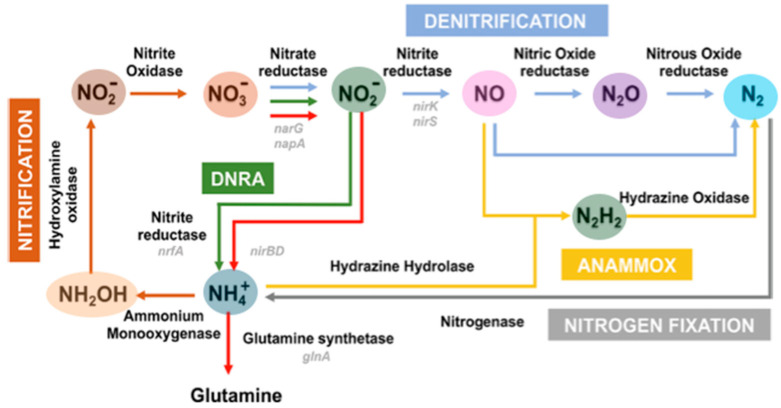
Major pathways of the nitrogen cycle in bacteria. Denitrification in blue, nitrification in brown, nitrogen fixation in grey, anammox (conversion of ammonia (DNRA) in green, nitrogen assimilation into hydrazine) in gold yellow, dissimilatory nitrate reduction to ammonium in red, while some genes encoding enzymes important in denitrification, DNRA, and nitrogen assimilation are highlighted in light grey. Nitrate reductase genes: narG and napA; Cu-containing nitrite reductase genes: nirK and nirS; nitrite reductase: nrfA and nirBD; and glutamine synthetase regulation gene: gInA. Adapted from [22].

The major steps are listed in Figure 1: Biological Nitrate reduction refers to microbial processes that convert NO_3_^−^ into nitrogen-containing compounds, often in environments with low oxygen availability. There are two primary types: (i) dissimilatory nitrate reduction to ammonium (DNRA) reduces NO_3_^−^ to NO_2_^−^ and then to NH_4_^+^ and (ii) denitrification uses four different metalloenzymes in four sequential steps, reducing NO_3_^−^ to NO_2_^−^, and sequentially to nitric oxide (NO), nitrous oxide (N_2_O), and, finally, nitrogen gas (N_2_) [6,19,20,21,22,23]. Both processes play crucial roles in the nitrogen cycle, influencing soil fertility, water quality, and atmospheric nitrogen balance.

Biological nitrite reduction is a microbial process in which NO_2_^−^ is converted into other nitrogenous compounds, depending on the biological pathway involved. This process occurs in both aerobic and anaerobic environments. Two primary pathways are described: (i) assimilatory nitrite reduction converts NO_2_^−^ into the NH_4_^+^ that is incorporated into organic molecules (e.g., amino acids and proteins) rather than converted it into gaseous nitrogen products. DNRA bacteria are involved in this process: (i) dissimilatory nitrite reduction is not used for biomass synthesis but for energy maintenance (respiration). Two distinct pathways exist in the denitrification reduction of NO_2_^−^ to NO: copper-containing Nitrite reductase (Cu-NiR and NirK) [6,19,22] and cytochrome *cd***_1_** Nitrite reductase (NirS). Reduction of NO_2_^−^ to NH_4_^+^ can be a respiratory process (the enzyme is a pentaheme cytochrome c) or a dissimilatory process (using a siroheme/iron–sulfur containing enzyme) [13].

Nitrite can be a source of NO, which is relevant in its maintenance under conditions of hypoxia/anoxia [24], as an alternative to NOS-catalyzed NO formation. Numerous metalloproteins with well-established functions are able to reduce nitrite to NO, and such examples are (i) the molybdenum-containing enzymes Xanthine oxidase/Xanthine dehydrogenase (XO/XD), Aldehyde Oxidase (AO), Sulfite Oxidase (SO), and Mitochondrial Amidoxime reducing component [24,25,26], as well as (ii) a growing number of heme-containing proteins (i.e., hemoglobin and myoglobin) [27,28,29,30].

In this review, a comparison is made between nitrate reductase and nitrite oxidation processes, which is supported by available structures and mechanistic proposals regarding various molybdenum-containing enzymes that play a key role in these processes. A challenging question, due to the structural similarities of Nitrate reductases and Nitrite oxidoreductases, is the understanding of the regulation of nitrate and nitrite interplay.

## 2. Redox Chemistry of N-Cycle

The N-cycle relies on the inter-conversion of N-species between different oxidation states spanning from +V to -III, (NO_3_^−^(+V), NO_2_^−^(+III), NO (+II), N_2_O (+I), N_2_ (0), NH_2_OH (-I), N_2_H_4_ (-II), and NH_3_ (-III)), driven by a series of redox reactions [6,22,23,31]. The majority of these transformations are catalyzed by microbial enzymes, including nitrogen fixation, nitrification, denitrification, anammox, and dissimilatory nitrate reduction to ammonium (DNRA) [4,32], involving specialized metalloenzymes such as Nitrogenase, [11,33] Nitrate reductases [6,23,30], Nitrite oxidoreductase [4,34], and Nitrous oxidoreductase [19,22]. The redox chemistry is associated with redox potentials (standard reduction potentials, E^0^, measured in volts at pH 7) and free energy (ΔG^0^) [35,36], which play a key role in understanding microbial nitrogen cycling and thermodynamic feasibility [11,13,23,37,38,39,40,41,42,43,44].

**Figure 2:**
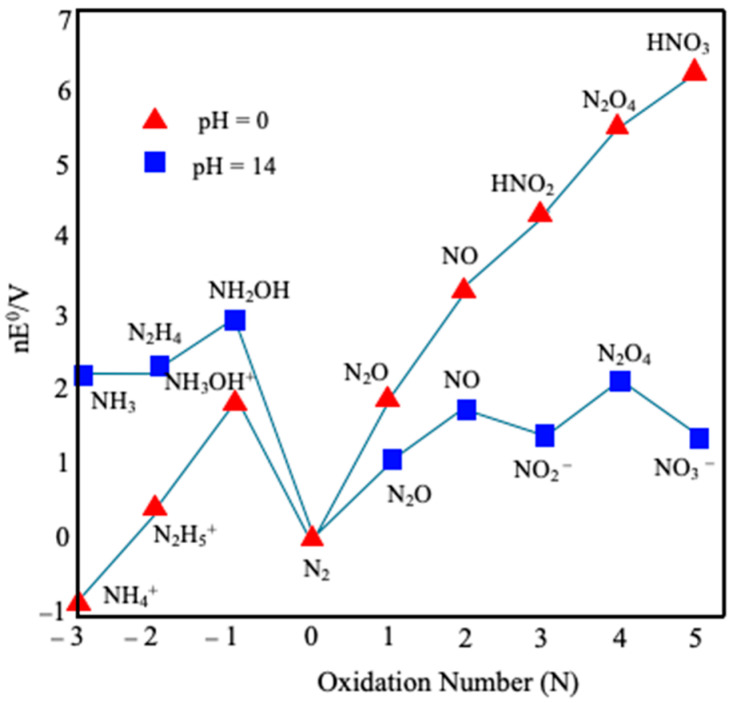
Frost diagram of N-elements at two different pHs, 0 and 14. Adapted from [36].

The thermodynamic relationship between inorganic N-species can be generally described by two diagrams, Latimer and Frost diagrams [35,36,37,38]. However, the Frost diagram allows for easier comprehension than the Latimer diagram. The Frost diagram, shown in Figure 2, highlights the complex redox relationship between various hydrido and oxo nitrogen species, as depicted in a representation of reduction potentials (E^0^) and thermodynamic stabilities (ΔG^0^) of different oxidation states of N-element in aqueous solution as a function of pH [36,38].

This diagram emphasizes that N_2_ and NH_3_ stand up as the most thermodynamically stable among nitrogen compounds under standard conditions, being the expected final products, as shown in the following Reactions (R1) and (R2):

2NO_3_^−^ +12H^+^ + 10e^−^ → N_2_ + 6H_2_O *E*^0^ = 1.17 V (vs. SHE) (R1)

NO_3_^−^ + 9H^+^ + 8e^−^ → NH_3_ + 3H_2_O *E*^0^ = −0.12 V (vs. SHE) (R2)

**Figure 3:**
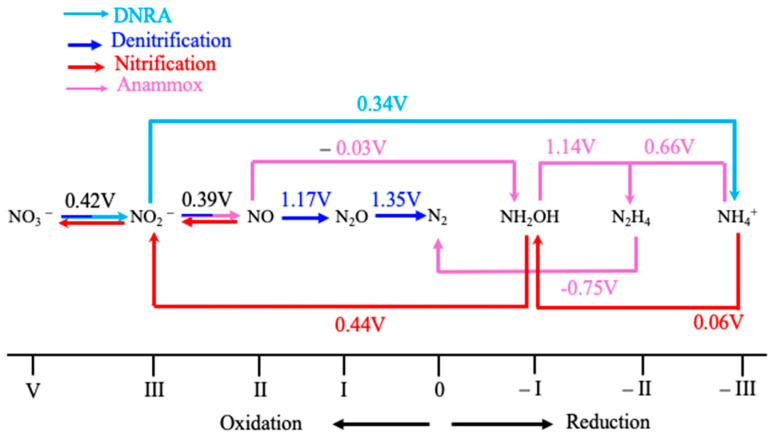
The diagram represents the oxidation states of various N-elements that accept or donate electrons, contributing to electron flow in the N-cycle by microbial enzymes. Standard redox potential at pH 7. Modified from [45].

The study of thermodynamics in the N-cycle is crucial for understanding the energy cost, redox gradient, and electron flow between N-species, and evaluates the microbial impact on the global N-distributions [39,40,41,42,43,44]. Here, the key reactions and their respective E**^0^** and ΔG^0^ are tabulated in Table 1 and Table 2, respectively, and a correlation diagram is represented in Figure 3. The N-biocycle is attributed to various nitrogen transforming pathways such as nitrification, denitrification, anammox, DNRA, and nitrogen fixation (Figure 3). Microorganisms use specialized metalloenzymes that perform these redox reactions involving inorganic nitrogen species with different oxidation states ranging from −3 to +5.

The correlation diagram (Figure 3) shows the electron flow in various nitrogen-transforming pathways formed by microbial enzymes. Indeed, ammonia is oxidized to nitrate through the nitrification pathway (NH_4_^+^ → NO_2_^−^ → NO_3_^−^), and then nitrate is converted to N_2_ gas through the denitrification pathway (NO_3_^−^ → NO_2_^−^ → NO → N_2_O → N_2_) or anammox pathway (NO_2_^−^ + NH_4_^+^ → N_2_) (Figure 3) [37,38,39,40,41,42]. These nitrogen-transforming pathways involve many reductive and oxidative reactions that are associated with redox potentials (E^0^), as shown in Table 1.

Table 2 shows that the negative value of ΔG^0^ is associated with microbial pathways involving interconversion between N-species in the N-cycle. In the anammox and nitrification pathways, NH_3_ is oxidized to N_2_ (Equation (1)) or NO_3_^−^ (Equation (2)), respectively. Similarly, in the denitrification pathway, NO_3_^−^ is reduced to NO_2_^−^, followed by NO, N_2_O, and N_2_ (Equation (3)). These reactions are thermodynamically favorable due to the negative ΔG^o^ value. In Equation (4), the reduction of NO_3_^−^ to NH_3_ is a two-step process where NO_3_^−^ is first reduced to NO_2_^−^, followed by reduction to NH_4_^+^. The reduction of N_2_ in nitrogen fixation is associated with negative ΔG^0^, suggesting it is thermodynamically favorable (Equation (9)). In Equations (5)–(10), all reactions show the negative ΔG^o^ values, indicating they are thermodynamically feasible. Moreover, these values indicate that denitrification (nitrate/nitrite reduction) is a highly favorable electron-accepting process, while ammonia oxidation is less favorable but still proceeds in aerobic environments. Overall, all reactions are thermodynamically feasible according to their redox potentials and free energy.

## 3. Oxygen Atom Transfer in the Nitrogen Cycle: Control of Active and Inactive Sites of Enzymes Involved—A General Mechanism

**Figure 4:**
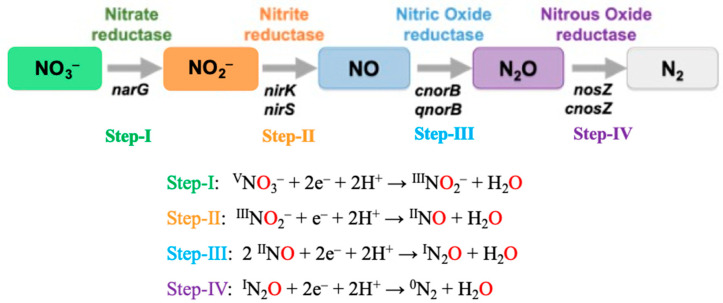
The transformation of NO_3_^−^ to N_2_ via NO_2_^−^, NO, and N_2_O pathways by selective metalloenzymes in each step.

All reactions involved in the stepwise transformations occurring in the denitrification and nitrification pathways share a common oxygen atom transfer (OAT) mechanism [6,25,27,30]. In denitrification, the first reaction converts nitrate to nitrite by Mo-dependent periplasmic nitrate reductase (NAP) or membrane-bound nitrate reductase (NAR) [23] (even the recently shown reversibility of nitrite to nitrate AOT ([46], see below), with copper and iron catalyze as subsequent steps. In the next step, the reduction of NO_2_^−^ to NO is catalyzed by Mo-containing nitrite reductases [28,30], a Cu-containing Nitrite reductase (nirK) [47], and a cytochrome *cd*_1_-containing Nitrite reductase (nirS) [48], which are functionally similar but structurally distinct (Figure 4). The reduction of NO to N_2_O is catalyzed by heme or nonheme nitric oxide reductase (NOR) encoded by diverse nor genes [49,50].

The conversion of NO to N_2_O is a unique and highly specific reaction that forms an N-N bond by iron/copper-containing Nitrous oxide reductase (N_2_OR) [19,22] (Figure 4). The concept of active and inactive catalytic metal sites is well documented throughout these processes. Mechanistic strategies observed in Nitrate reductases (NAR) share similarities with other molybdenum-containing enzymes, such as Dimethyl sulfoxide reductase [23,25]. The substrate oxygen abstraction mechanism leads to the nitrate-to-nitrite conversion via oxygen atom transfer to the metal center, with water serving as the final acceptor of the abstracted oxygen atom [6,48].

For enzymes lacking an exchangeable oxygen ligand, such as *Desulfovibrio desulfuricans* and *Cupriavidus necator* periplasmic NAR, an alternative pathway known as the sulfur shift mechanism has been proposed [51,52,53]. In this mechanism, nitrate reaches the inactive oxidized Mo = S center, triggering the insertion of a sulfur atom into the Mo–S bond, yielding an active (Cys)S–S–Mo core. This interaction creates a binding site for nitrate, converting the enzyme from an inactive to an active form. The formation of a (Cys)S–S–Mo–ONO_2_ intermediate follows, leading to cleavage of the O–NO_2_ bond, releasing nitrite, and catalyst regeneration for the next catalytic cycle. The sulfur shift mechanism enables an enzyme with a fully coordinated metal center to become catalytically active, allowing for controlled regulation of enzyme function [51].

A similar activation event occurs in copper Nitrite reductase (CuNiR) [54]. Two distinct copper sites exist: an electron transfer site with full coordination and a catalytic site with a vacant site that binds the substrate [54,55]. No activation is required for CuNiR as its catalytic site is already in a ready form. However, in cytochrome *cd*_1_ Nitrite reductase (cd1NiR), activation is necessary to convert it from an inactive to an active state [6,56]. This occurs through conformational and ligation alterations of hemes *c* and *d*_1_, with the latter becoming high spin and ready to bind the substrate. A similar understanding applies to pentaheme cytochrome Nitrite reductase (*cc*NiR), where a single high-spin heme defines the reaction site, and to N_2_O reductase, where one of the copper atoms has a lower coordination state [6,13,57,58].

**Figure 5:**
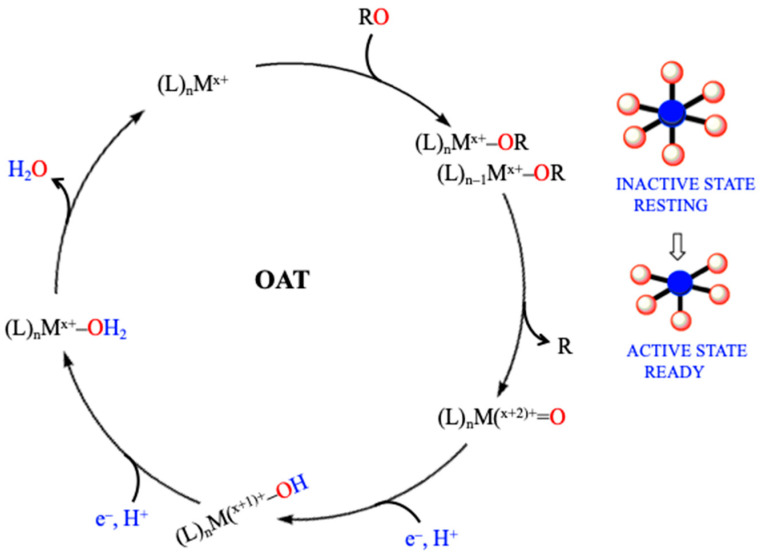
A general substrate oxygen abstraction mechanism leads to the RO to R (nitrate-to-nitrite/nitrite-to-nitric oxide) conversion via oxygen atom transfer to the metal center. L: ligand; n: number of ligands; M: metal; x = oxidation state; and OAT: oxygen atom transfer. Modified from [59].

These observations support a general mechanism for all OAT steps in the nitrogen cycle (Figure 5). This universal mechanism considers the resting states of enzymes, where a coordination site is either available for substrate binding or requires a conformational activation step to create one. Substrate binding then facilitates oxygen removal, followed by electron (2e^−^) and proton (2H^+^) transfer steps. These steps make oxygen more easily incorporated into H_2_O, therefore restoring the catalytic site for further reactions. The interplay of active (ready) and inactive states (unready) in these enzymes highlights the dynamic regulation of nitrogen cycle transformations through metal-centered catalysis [6,59].

## 4. Nitrate Reduction and Nitrite Oxidation

Mononuclear molybdenum (Mo) containing enzymes are a fascinating group of metalloenzymes that play essential roles in various biological processes [25,26,60]. Molybdenum is a trace element, but vital because it forms part of the active site in enzymes involved in electron and atom transfer. Almost all molybdenum enzymes (in humans and many other organisms) require a special cofactor called molybdopterin [61,62,63]. The molybdenum ion is bound to this organic molecule, which helps position and stabilize it for catalysis. Major classes of molybdenum enzymes include Xanthine Oxidase/Dehydrogenase that converts xanthine into uric acid in purine metabolism, which is important in uric acid production and oxidative stress, and is also linked to gout and kidney stones; Aldehyde Oxidase (AO) converting aldehydes to carboxylic acids, involved in drug metabolism with a broad substrate specificity; Sulfite Oxidase that oxidizes sulfite into sulfate, a critical process for detoxifying sulfites (preservatives in food) and related genetic deficiency that leads to severe neurological disorders; and Dimethyl Sulfoxide Reductase (DMSOR) family (in bacteria) using various substrates, including dimethyl sulfoxide (DMSO), found in anaerobic respiration [25,26,60,61,62,63,64,65,66,67].

Related to the N-cycle, Nitrate Reductase (in plants and bacteria) and Nitrite oxidoreductase are crucial Mo-enzymes in the nitrate/nitrite interplay and are the focus of the review [6]. Nitrogenase catalyze is one of the most important reactions on Earth: the conversion of atmospheric nitrogen (N_2_) into ammonia (NH_3_), or so-called biological nitrogen fixation [11,68]. Plants cannot use atmospheric nitrogen (N_2_) directly, but Nitrogenase allows certain bacteria and archaea (like Rhizobium, Azotobacter, and cyanobacteria) to “fix” nitrogen, turning it into a usable form. This ammonia becomes part of the nitrogen cycle, feeding ecosystems. Nitrogenase is a complex enzyme system, and the MoFe protein component (Molybdenum–Iron protein) contains the unique FeMo-cofactor (7 Fe, 1 Mo, 9 S, plus a central C atom) and P-cluster ([8Fe-7S]) that transfers electrons to the FeMoco [11,68]. There are also alternative forms of nitrogenase: V-nitrogenase [69] uses vanadium instead of molybdenum, and Fe-only nitrogenase is used when Mo is scarce [70].

Nitrate reduction to nitrite and nitrite oxidation to nitrate are two interrelated processes in the nitrogen cycle, representing opposite yet complementary reactions [6,23,46,71,72]. Nitrate reductases (NAR, NAS, and NAP) are reductive enzymes that use nitrate as an electron acceptor, while nitrite oxidoreductase (NXR) is an oxidative enzyme that uses nitrite as an electron donor [23,46,71,72,73,74,75]. Evolutionary and functional connections reveal that both processes involve molybdenum cofactors (Moco) and iron–sulfur clusters, which are crucial for their redox activity [72,73]. Although these enzymes catalyze reactions in opposite directions, they likely evolved from a common ancestral enzyme, adapting to distinct roles in nitrogen metabolism.

Nitrate reduction occurs in both biological and chemical contexts. In assimilatory nitrate reduction, organisms reduce nitrate to nitrite and then to ammonia for incorporation into biomolecules [6,23]. In dissimilatory nitrate reduction (denitrification and DNRA—dissimilatory nitrate reduction to ammonium), nitrate is reduced to nitrite and further to gaseous nitrogen (N_2_) or ammonium, often under anaerobic conditions. Common bacteria involved are *Escherichia coli* (DNRA) and *Pseudomonas* sp. (denitrification) [6,23].

Nitrite oxidation to nitrate (nitrification) is an aerobic process where nitrite-oxidizing bacteria (NOB) convert nitrite into nitrate. This step is critical for wastewater treatment and soil nitrogen cycling [76,77].

**Why “Two Sides of the Same Coin”?** Both processes regulate nitrogen transformation, maintaining balance in the nitrogen cycle. They influence nitrogen availability in ecosystems, affecting plant growth, microbial activity, and greenhouse gas emissions. While nitrate reduction predominantly occurs in anaerobic or low-oxygen environments, nitrite oxidation thrives in oxygenated conditions, ensuring dynamic equilibrium [6,23,78].

### 4.1. Nitrate Reduction (NO_3_^−^ → NO_2_^−^)

The periplasmic nitrate reductase catalytic subunit (NAP-A) is a Moco-containing enzyme that reduces nitrate to nitrite [46,78,79,80,81,82]. Some DMSO reductase (DMSOR) family enzymes catalyze reversible reactions, and recent studies have also demonstrated that *Campylobacter jejuni* NAP-A can also oxidize nitrite to nitrate, marking the first evidence of its bidirectional activity [46].

#### 4.1.1. Enzymatic Machinery

There are distinct nitrates located in different subcellular compartments, used for both dissimilatory and assimilatory processes: (i) membrane-bound cytoplasm-faced respiratory NAR; (ii) periplasmic NAR that contributes to proton motive force generation; and (iii) cytoplasmic assimilatory NAR involved in nitrogen assimilation [23,52,53,71,72,83,84,85,86,87,88,89,90,91].

**Figure 6:**
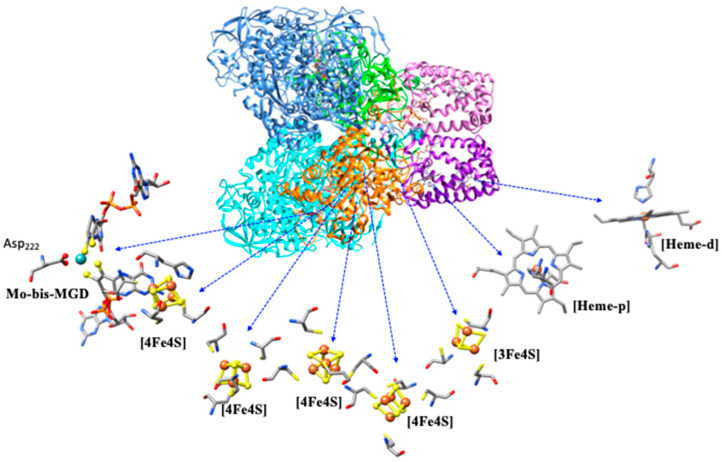
Crystal structure of a dimer of heterotrimer (ribbon of monomer-1/monomer-2: cyan/corn blue, orange/lime-green, and purple/pink) Nitrate Reductase, NAR-GHI, from *Escherichia coli* (PDB: 1Q16). Highlighted at the active site. Mo: cyan ball, Fe: red ball, and S: yellow ball.

Due to their distinct biological roles and locations, these enzymes have different subunit organizations and cofactor compositions. For example, the respiratory enzyme *E. coli* NAR (Nar-GHI), encoded by the following genes: narG, narH, and narI—a dimer of heterotrimers (αβγ)_2_: (i) NarG (cytoplasmic subunit) contains the molybdenum center and one [4Fe-4S] cluster; (ii) NarH (electron transfer subunit) has one [3Fe-4S] and three [4Fe-4S] clusters involved in electron transfer from the membrane quinol pool to the molybdenum center; and NarI (membrane-bound quinol-oxidizing subunit) harbors two b-type hemes [72,90] (Figure 6). In contrast, the periplasmic NAR from *D. desulfuricans* (napA gene) is monomeric, with only one [4Fe-4S] cluster in addition to the molybdenum center. Similarly, *Cupriavidus necator* produces a dimeric periplasmic nitrate reductase (NAP-AB), in which NAP-A harbors a molybdenum center and an iron–sulfur cluster, while NAP-B contains two heme c groups.

#### 4.1.2. Enzymatic Mechanism

Despite catalyzing the same reaction (two-electron reduction of nitrate to nitrite at the molybdenum center), these enzymes exhibit significant differences in their molybdenum coordination spheres [23,71,72]. In the respiratory membrane-bound in NAR, the molybdenum atom is coordinated by an aspartate residue in a monodentate or bidentate fashion, which may correspond to oxidized and reduced states, respectively [72]. In the periplasmic NAR from *D. desulfuricans* and *C. necator*, the molybdenum atom shows a different coordination, with a cysteine sulfur atom and a terminal sulfo group, forming a partial disulfide bond. *E. coli* and *Rhodobacter sphaeroides* periplasmic NAR coordinate the molybdenum atom with a cysteine sulfur atom and a terminal hydroxyl group [50,52,72,81]. The least studied among these enzymes is cytoplasmic assimilatory NAR, but it is likely coordinated by a cysteine residue.

**Figure 7:**
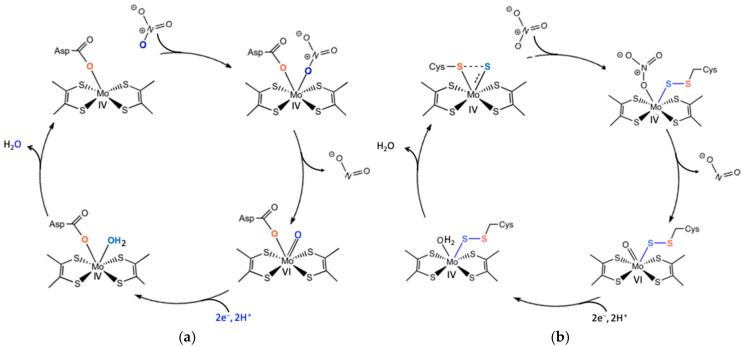
Schematic description of the proposed mechanism for nitrate reduction at the membrane-bound NAR active site (**a**) and sulfur shift at periplasmic NAR active site (**b**). Modified from [79,81].

Mechanistic aspects are parallel with other molybdenum enzymes, since similar mechanistic strategies have been observed in other molybdenum-containing enzymes, such as DMSOR (dimethyl sulfoxide reductase) and formate dehydrogenase (FDH) [25,89]. DMSOR and NAR share oxygen abstraction mechanisms. The molybdenum center cycles between an oxo-labile Mo^VI^ core and a reduced Mo^IV^ core, with water as the final destination for the abstracted oxygen atom. Periplasmic NAR from *E. coli* employs a mechanistic strategy in which nitrate binds to a Mo^V^ oxidation state rather than Mo^IV^. Upon reduction, the nitrate-to-nitrite transformation proceeds via oxygen atom transfer to the metal (Figure 7) [6,23,92,93,94].

The sulfur shift mechanism was an alternative proposed for the enzymes lacking an exchangeable oxygen ligand (e.g., *D. desulfuricans* and *C. necator* periplasmic NARs) [51,81,95,96]. Nitrate reaches the inactive oxidized Mo = S center, triggering insertion of the sulfur atom into the Mo–S bond, yielding an active (Cys)S–S–Mo core. In this way, a binding site is created for nitrate, forming a (Cys)S–S–Mo–ONO_2_ intermediate. O–NO_2_ bond cleavage is the next step, and nitrite is released, regenerating a Mo^VI^ oxo-labile core for the next catalytic cycle (Figure 7) [51,81,90,91,92,93,94,95,96]. This sulfur shift mechanism allows an enzyme with a fully coordinated metal center to become catalytically active, enabling controlled regulation of enzyme function.

### 4.2. Nitrite Oxidation (NO_2_^−^ → NO_3_^−^)

Nitrite oxidoreductase (NXR) is the key enzyme in the oxidation of nitrite (NO_2_^−^) to nitrate (NO_3_^−^) completing the nitrification process, a crucial step in the nitrogen cycle, and Nitrite oxidoreductase (NXR) is the key enzyme [6,73,97,98]. This process plays a critical role in soil and aquatic ecosystems, maintaining nitrogen balance and affecting agriculture and wastewater treatment, since nitrate accumulation can lead to eutrophication [76,97]. This enzyme is primarily found in nitrifying bacteria, such as *Nitrobacter*, *Nitrospira*, and *Nitrococcus* [34]. This oxidation reaction is highly exergonic, meaning that it releases energy and is not easily reversible under normal physiological conditions.

This process usually occurs under aerobic conditions, where nitrite acts as an electron donor and oxygen serves as an electron acceptor. Conversely, under anaerobic conditions, certain microorganisms can use NXR in reverse, reducing nitrate (NO_3_^−^) back to nitrite (NO_2_^−^) [4,73,99]. The direction of the reaction depends on environmental factors such as oxygen availability, redox potential, and the metabolic needs of the microorganism. This enzymatic flexibility is essential for microbial adaptation to different ecological niches and contributes significantly to global nitrogen cycling.

#### 4.2.1. Enzymatic Machinery

NXR is a large multi-subunit mononuclear molybdenum-containing enzyme (from the DMSO family [25]), and the dissimilatory oxidation of nitrite to nitrate takes place at the molybdenum center [73]. NXR (encoded by *nxr* genes) is a membrane-bound protein, linked to the electron transport chain for ATP generation, that can be divided into two groups based on subcellular localization: (i) periplasmic-facing the periplasmatic side of the cytoplasmic membrane (i.e., *Nitrospira, Nitrospina*), and (ii) cytoplasmic-facing enzymes anchored on the cytoplasmic side (*Nitrobacter* and *Nitrococcus*) [6,34,73,97,98,99]. Despite its vital role, detailed structural information on NXR has been limited [99,100,101]. Recent cryo-EM and X-ray crystallography studies have resolved *Kuenenia stuttgartiensis* NXR structure, a heterotrimer, providing insights into its architecture and function [73]. The molybdenum center, where nitrite oxidation occurs, is coordinated by two pyranopterin cofactors and amino acid residues such as cysteine, as in NarG. Additionally, an oxo group at the molybdenum center is thought to participate in the reaction, transferring an oxygen atom to nitrite to form nitrate. The Mo-site cycles between oxidation states (Mo^VI^/Mo^IV^) during the redox process, enabling electron transfer to Fe-S clusters and heme groups. Structurally, NXR is known to form densely packed tubule structures on the membrane surface, with lengths extending to hundreds of nanometers [102,103]. The tubules are formed by the arrangement of head-to-tail dimers of heterotrimers of NXR, and they exhibit nitrite oxidation activity. The purpose of NXR tubule formation remains unclear. The interest in enzymes forming tubules, or similar assemblies, has revealed potential roles, including stabilization, partner binding, and specificity control [104,105].

**Figure 8:**
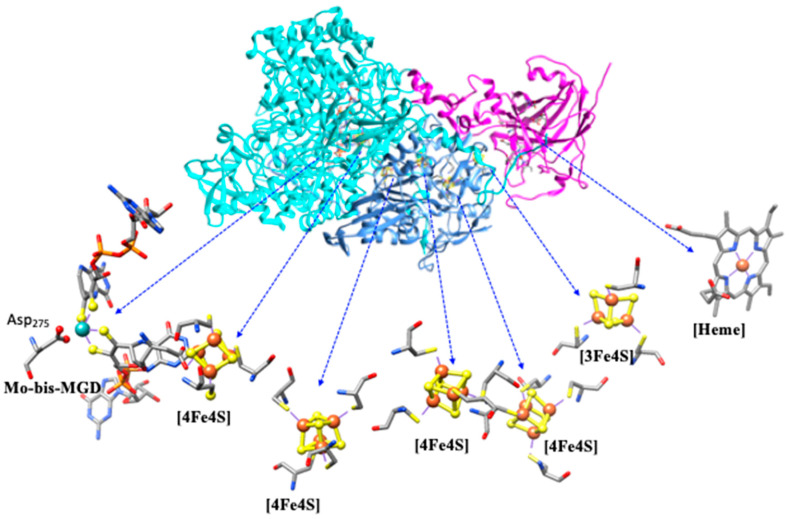
Crystal structure of heterotrimer of Nitrite oxidoreductase (NXR) from the anammox bacterium *Kuenenia stuttgartiensis* (PDB: 7B04) (ribbon: cyan, corn-blue, and magenta).

The *Nitrobacter hamburgensis* NXR, a cytoplasmic-facing enzyme, is also a heterotrimer composed of a catalytically active αβ-complex (~115 and 65 kDa) associated with a membrane c-type heme γ-subunit (~32 kDa). The *Nitrobacter hamburgensis* NXR β-subunit (encoded by *nxrB*) displays a cysteine distribution identical to that of *E. coli* NAR-H (with a 45% sequence identity) (Figure 8) [73].

Accordingly, it likely contains three [4Fe−4S] clusters and one [3Fe−4S] cluster. The *Nitrospira moscoviensis* NXR, a periplasmic-facing enzyme, also consists of a catalytically active αβ-complex (~130 and 46 kDa), and a transmembrane γ-subunit, which was hypothesized to facilitate electron transfer between the β-subunit and the electron transport chain, which has not yet been identified. For a periplasmic-facing enzyme, the electrons from quinol oxidation do not necessarily need to pass back across the membrane, so a membrane-bound heme subunit may not be required. Similarly, four Fe/S-binding motifs have been identified in the β-subunits of *Nitrococcus* and *Nitrospira*, being proposed, by the similarities with NarH, to facilitate electron transfer from the α-subunit (where nitrite oxidation occurs) to the γ-subunit or directly to the membrane electron transport chain. The α-subunit (encoded by *nxrA*) also shows significant similarity to the C-terminal sequences of *E. coli* NarG and contains one Fe/S center and one molybdenum center.

#### 4.2.2. Enzymatic Mechanism

The NXR-catalyzed the oxidation of nitrite (NO_2_^−^) to nitrate (NO_3_^−^), but the detailed catalytic mechanism of it remains poorly understood. Although the 3D structure including Mo-active site, iron–sulfur clusters, and heme groups of NXR has been reported in the literature [73], substrate-bound 3D structures or intermediates trapped during the catalytic cycle are yet absent, lacking the resolution of precise substrate-binding mode and transition states. Interestingly, NXR is able to catalyze nitrite oxidation and nitrate reduction as well [4,17,34,74,99,106]. So, bi-direction electron flow in the catalytic cycle of NXR remains unclear. In addition, all redox partners are connected to each other to involve the electron relay in the catalytic cycle, but their direct role in nitrite oxidation remains speculative. Moreover, the role tubule structures in the activity of NXRs remains yet unclear. Analogous enzymes, NARs, have been extensively characterized using spectroscopic methods, providing detailed mechanistic insights [51,81,90,91,92,93,94,95,96]. Such studies are absent for NXRs, creating a significant knowledge gap for complete understanding of its role in nitrite oxidation.

**Figure 9:**
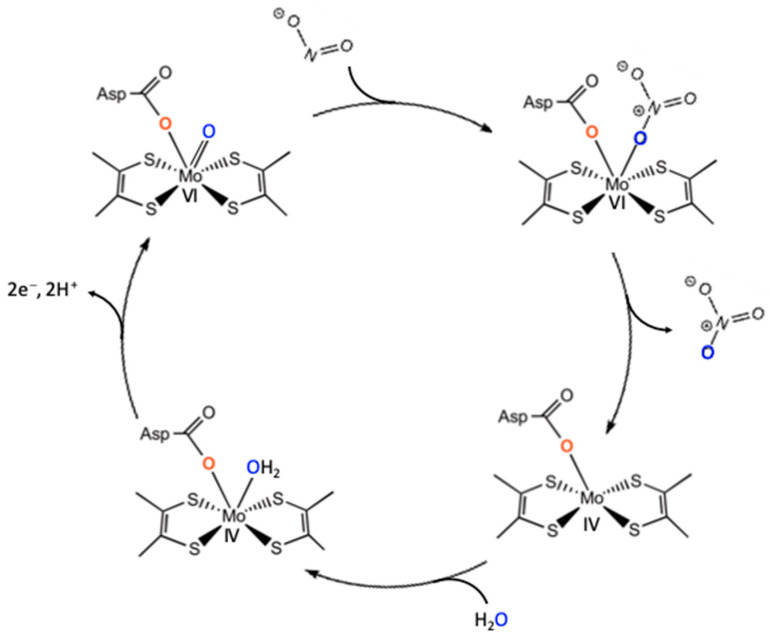
Probable mechanism of the oxidative transformation of nitrite to nitrate by NXR.

As in other molybdenum-containing enzymes, the oxygen atom in the resulting nitrate originates from water rather than molecular oxygen (Figure 9). The molybdenum center likely plays a key role in mediating oxygen atom transfer [73]. Further studies are required to elucidate the structure and mechanistic details of both cytoplasmic- and periplasmic-facing NXR enzymes. Being metabolically versatile, they can catalyze both nitrite oxidation and nitrate reduction, alternating between aerobic nitrite oxidation to anaerobic growth via dissimilatory nitrate reduction. Depending on the species, *Nitrobacter* can use pyruvate or hydrogen as an electron donor and *Nitrospira* can use hydrogen. However, the *Nitrobacter hamburgensis* NXR has been shown to catalyze nitrate reduction in vitro, while the *Nitrospira* enzyme does not. It remains unknown whether these bacteria use the same enzyme for both processes or synthesize a different protein de novo.

### 4.3. How Similar Are Nitrate Reductases and Nitrite Oxidoreductases

Understanding the interplay between microbial processes that drive nitrogen transformations is crucial for comprehending the complexity and resilience of biogeochemical cycles. Among these, nitrate reduction and nitrite oxidation stand out as key mechanisms that not only mediate the conversion of nitrogen compounds but also maintain the stability of the nitrogen cycle across diverse ecosystems. Their functional inter-dependence, despite occurring under contrasting redox conditions, underscores the rationale behind the phrase “**two sides of the same coin**” [6,23,24,46,73]. Nitrate reduction typically occurs under anaerobic or low-oxygen conditions, while nitrite oxidation is favored in oxygenated environments. Together, they exert a profound influence on nitrogen availability, shaping plant productivity, microbial activity, and the flux of greenhouse gases. Recognizing their complementary roles highlights the importance of integrated approaches in studying nitrogen dynamics and reinforces the need for continued research into the environmental factors that modulate these processes.

**Figure 10:**
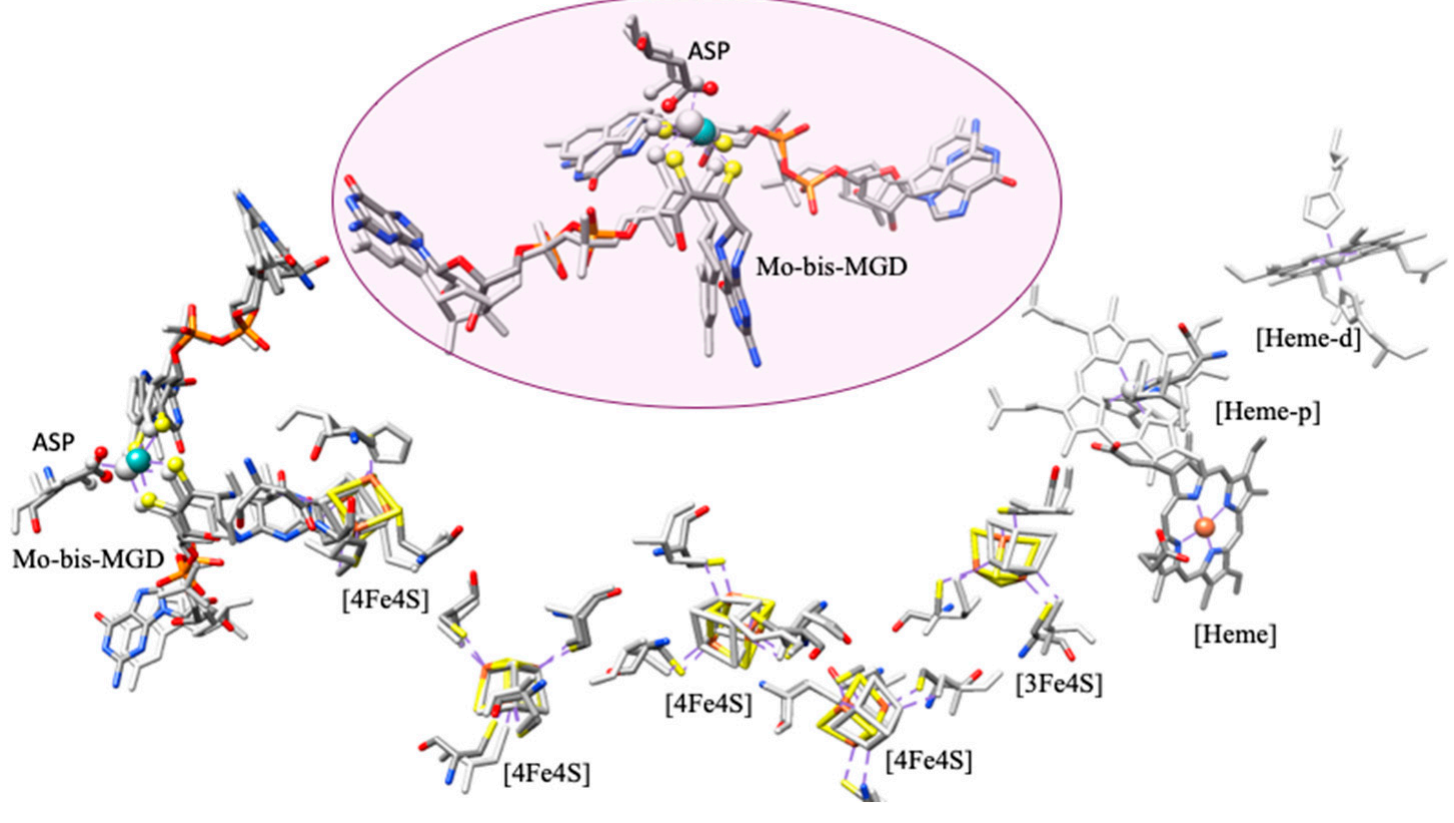
Superimposition of active sites with Fe/S clusters and heme of Nitrate Reductase A, NAR-GHI, from *Escherichia coli* (PDB: 1Q16) (atoms: grey color), and Nitrite oxidoreductase (NXR) from the anammox bacterium *Kuenenia stuttgartiensis* (PDB: 7B04) (atoms: color).

**Figure 11:**
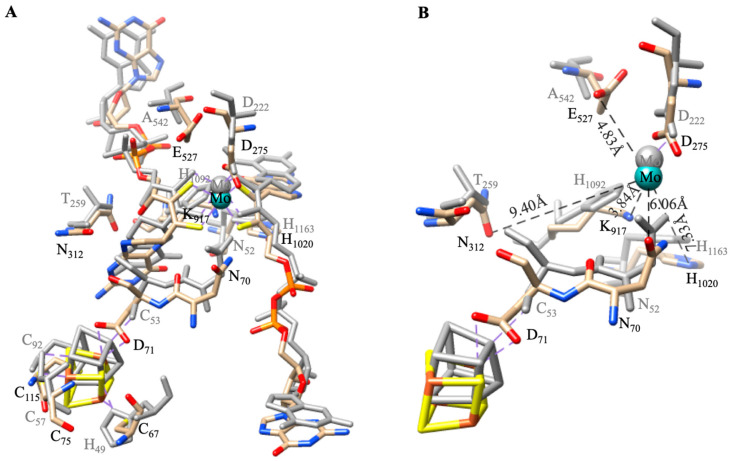
Superimposition of active site structures of Nitrate Reductase A, NAR-GHI, from *Escherichia coli* (PDB: 1Q16) (atoms: grey color) and Nitrite oxidoreductase (NXR) from the anammox bacterium *Kuenenia stuttgartiensis* (PDB: 7B04) (atoms: color). The vicinity amino acids at the active site are highlighted (**A**). For clarity, the molybdopterin guanine dinucleotide (MGD) is omitted, and distances are highlighted (**B**).

The active site structure and [Fe-S] clusters of Nitrite oxidoreductase (NXR) [73] are very similar to those of Nitrate Reductase, NAR-GHI, which is shown by superimposition of both structures (Figure 10) [72,90]. However, some striking differences are found between NAR and NXR (Figure 11). In both, the proximal [4Fe-4S] cluster connects to the Mo-active site through the dipeptide amino acid chain, Asn_70_Asp_71_ in NXR and Asn_52_Cys_53_ in NAR. The [4Fe-4S] is coordinated by 3-Cys and 1-Asp_71_ in NXR and 3-Cys and 1-His_49_ in NAR. This variation may affect the redox potential of [4Fe-4S] in NAR and NXR, which has functional importance. In addition, in the vicinity of the active sites of NXR/NAR, some amino acids like Glu_527_/Ala_542_ (E/A), Asn_312_/Thr_259_ (N/T), Lys_917_/His_1092_ (K/H), and Asp_71_/Cys_53_ (D/C) are different, which may tune the activity, suggesting the different activity between NAR and NXR instead of the same active site [68,69,86]. Indeed, *C. jejuni* NapA contains a conserved lysine residue situated between the Mo-cofactor and [4Fe–4S] cluster. This lysine bridges two redox centers through H-bonding, potentially playing a crucial role in facilitating the intramolecular electron transfer essential for NapA activity [107].

The reversibility of NAP-A-catalyzed nitrate reduction challenges the conventional understanding of nitrate and nitrite redox processes and positions NAP-A as functionally comparable to nitrite oxidoreductases (Nir). Traditionally, nitrate reductases like NAP-A have been studied primarily for their role in reducing nitrate (NO_3_^−^) to nitrite (NO_2_^−^), a unidirectional process assumed to be distinct from the oxidation reaction carried out by Nir enzymes. However, recent findings demonstrate that NAP-A is not only capable of reducing nitrate to nitrite but also catalyzes the reverse oxidation of nitrite back to nitrate [46]. This dual functionality suggests a bidirectional capability governed by oxygen atom transfer (OAT) mechanisms. Conversely, under anaerobic conditions, certain microorganisms can use NXR in reverse, reducing nitrate (NO_3_^−^) back to nitrite (NO_2_^−^) [73,99]. These findings not only expand the functional landscape of NAP-A but also blur the lines between nitrate reductases and nitrite oxidoreductases, emphasizing the mechanistic reversibility of redox enzymes traditionally thought to operate in one direction. Our proposed mechanistic scheme, supported by both experimental and computational data, underscores oxygen atom transfer as the rate-limiting step, further aligning NAP-A’s activity with that of Nir and redefining its role in microbial nitrogen cycling.

## 5. Conclusions

Nitrate reduction (NAR) and nitrite oxidation (NXR) constitute two fundamental yet functionally opposing branches of the nitrogen cycle. Although operating in reverse directions—reducing nitrate to nitrite versus oxidizing nitrite to nitrate—these processes are deeply interconnected and exemplify the chemical and evolutionary elegance of redox biology. Both are dependent on molybdenum cofactor (Moco)-based enzymatic catalysis, centered on a pyranopterin-coordinated molybdenum active site, and trace their origins to a shared ancestral redox machinery.

The mechanistic diversity observed among nitrate reductases—spanning respiratory, periplasmic, and assimilatory forms—demonstrates the remarkable adaptability of Mo enzymes in response to cellular and environmental demands. Similarly, NXR enzymes exhibit varied cellular localizations and orientations (cytoplasmic- vs. periplasmic-facing), yet converge on a conserved biochemical strategy. Notably, the structural parallels between cytoplasmic-facing NAR-GHI, periplasmic NAR, cytoplasmic anabolic NAR, and both cytoplasmic- and periplasmic-facing NXRs suggest a modular evolutionary design, optimized for directional electron transfer and efficient redox conversion between nitrate and nitrite.

From a redox perspective, these enzymes serve as central hubs in cellular electron flow—anchoring nitrogen metabolism within broader energy transduction networks. NAR typically functions in low-oxygen or anaerobic environments, serving as a terminal electron acceptor in respiration, whereas NXR operates in oxygen-rich niches, facilitating chemolithoautotrophic energy acquisition. Despite these environmental distinctions, both enzyme systems embody nature’s convergent solution to the challenge of nitrogen interconversion: a finely tuned redox platform leveraging molybdenum chemistry to sustain global nitrogen cycling.

Future directions should focus on elucidating the structural and spectroscopic nuances that govern substrate specificity, redox potential tuning, electron channeling within these enzyme families, and direct functional reversibility. A deeper mechanistic understanding will not only clarify their evolutionary trajectories but also enhance our capacity to model and manipulate nitrogen fluxes in both natural and engineered systems. As our knowledge of redox enzyme biochemistry advances, so too does our appreciation of the shared molecular logic underpinning seemingly divergent pathways—truly two sides of the same coin.

## Figures and Tables

**Table 1:** Redox potentials of various biochemical reactions involved in N-biocycle.

Pathways	Reactions	ReductionPotentials (E^0^) (at pH 7)
Assimilatory and dissimilatory nitrate reduction	**Nitrate Reduction** (NO_3_^−^ → NO_2_^−^) ^V^NO_3_^−^ + 2H^+^ + 2e^−^ → ^III^NO_2_^−^ + H_2_O	+0.42 V
Dissimilatory nitrate reduction to Ammonium (DNRA)	**Nitrate to Ammonium** (NO_3_^−^ → NO_2_^−^→ NH_3_) ^III^NO_2_^−^ + 8H^+^ + 6e^−^ → ^-III^NH_4_^+^ + 2H_2_O	+0.34 V
Denitrification	**Nitrite Reduction (NO_2_**^−^**→ NO, N_2_O, or N_2_)** **Several steps occur in denitrification:** **Nitrite to Nitric Oxide** (NO_2_^−^ → NO) ^III^NO_2_^−^ + 2H^+^ + e^−^ → ^II^NO + H_2_O	+0.39 V
	**Nitric Oxide to Nitrous Oxide** (NO →N_2_O) 2 ^II^NO + 2H^+^ + 2e^−^→ ^I^N_2_O + H_2_O	+1.17 V
	**Nitrous Oxide to Dinitrogen Gas** (N_2_O → N_2_) ^I^N_2_O + 2H^+^ + 2e^−^ → ^0^N_2_ + H_2_O	+1.35 V
Nitrification	**Nitrite Oxidation** (NO_2_^−^ → NO_3_^−^) ^III^NO_2_^−^ + H_2_O → ^V^NO_3_^−^ + 2H^+^ + 2e^−^	+0.42 V
	**Ammonia Oxidation** (NH_4_^+^ → NO_2_^−^) **Ammonia to Hydroxylamine** ^-III^NH_3_ + 1/2O_2_ + H^+^ + 2e^−^ → ^-I^NH_2_OH	+0.06 V
	**Hydroxylamine to Nitrite** (NH_2_OH → NO_2_^−^) ^-I^NH_2_OH + H_2_O → ^III^NO_2_^−^ + 5H^+^ + 4e^−^	+0.44 V
	**The overall oxidation of ammonia to nitrite**: ^-III^NH_3_ + 1/2O_2_ → ^III^NO_2_^−^ + H_2_O + H^+^	+0.34 V
Anammox	**Hydrazine to Nitrogen** (N_2_H_4_ → N_2_) ^-II^N_2_H_4_ → ^0^N_2_ + 4H^+^ + 4e^−^. ^II^NO + ^−III^NH_4_^+^ + 2H^+^ + 3e^−^ → ^−II^N_2_H_4_ + H_2_O	−0.75 V +0.126
Nitrogen fixation	**Nitrogen to Ammonium** (N_2_ → NH_3_) ^0^N_2_ + 6H^+^ + 6e^−^ → 2 ^-III^NH_3_	+0.09 V

Nitrate is a good electron acceptor and is readily reduced to nitrite in anaerobic conditions. Denitrification (nitrate/nitrite reduction) is a highly favorable electron-accepting process, while ammonia oxidation is less favorable but still proceeds in aerobic environments. This reaction is catalyzed by nitrite-oxidizing bacteria (NOB) such as *Nitrobacter* and *Nitrospira*.

**Table 2:** ΔG^o^ value of bio-chemical reactions involved in N-biocycle.

Reactions	ΔG^o^ (kJ/mol)	Equation
^-III^NH_4_^+^ + ^III^NO_2_^−^ → ^0^N_2_ + 2H_2_O	−358	(1)
2 ^-III^NH_3_ + 2O_2_ → ^IV^NO_3_^−^ + 3H_2_O	−349	(2)
2 ^IV^NO_3_^−^ + 2H^+^ + 5H_2_ → ^0^N_2_ + 6H_2_O	−1121	(3)
2 ^IV^NO_3_^−^ + 2H^+^ + 4H_2_ → ^-III^NH_4_^+^ + 3H_2_O	−591	(4)
^III^NO_2_^−^ + 2H^+^ + e^−^ → ^II^NO + H_2_O	−113.38	(5)
^III^NO_2_^−^ + ^−III^NH_4_^+^ → N_2_ + 2H_2_O	−357	(6)
^II^NO + ^−III^NH_4_^+^ + 2H^+^ + 3e^−^ → ^−II^N_2_H_4_ + H_2_O	−116.27	(7)
^-II^N_2_H_4_ → ^0^N_2_ + 4H^+^ + 4e^−^	−128.10	(8)
^0^N_2_ + 2H^+^ + 3H_2_ → 2 ^-III^NH_3_	−39	(9)
^-III^NH_4_^+^ + NO_2_^−^ → N_2_ + 2H_2_O	−119	(10)

## Data Availability

Data are contained within the article.

## Abbreviations

The following abbreviations are used:

Anammox	Anammox: Anaerobic Ammonium Oxidation
AO	Aldehyde Oxidase
Comammox	Complete Ammonia Oxidation
*cd*_1_NiR	cytochrome *cd*_1_ Nitrite reductase
*cc*NiR:	cytochrome *c* Nitrite reductase
Cu-NiR	Copper-containing Nitrite reductase;
*C. necator*	Cupriavidus necator
DNRA	Dissimilatory nitrate reduction to ammonium
DMSOR	Dimethyl Sulfoxide Reductase
*D. desulfuricans*	*Desulfovibrio desulfuricans*
*E. coli*	*Escherichia coli*
FDH	formate dehydrogenase
Moco	molybdenum cofactors
MGD	molybdopterin guanine dinucleotide
N-cycle	Nitrogen cycle
NXR	Nitrite oxidoreductase
NAP	periplasmic Nitrate reductase
NAR	respiratory Nitrate reductase
NAS	assimilatory Nitrate reductase
NOR	Nitric oxide reductase
N_2_OR	Nitrous oxide reductase
NOB	Nitrite-oxidizing bacteria
OAT	oxygen atom transfer
PDB	protein data bank
SO	Sulfite oxidase
XO/XD	Xanthine Oxidase/Xanthine Dehydrogenase
